# Small Renal Masses: Incidental Diagnosis, Clinical Symptoms, and Prognostic Factors

**DOI:** 10.1155/2008/310694

**Published:** 2009-01-21

**Authors:** F. M. Sánchez-Martín, F. Millán-Rodríguez, G. Urdaneta-Pignalosa, J. Rubio-Briones, H. Villavicencio-Mavrich

**Affiliations:** ^1^Servicio de Urología, Fundació Puigvert, C/Cartagena 340, 08025 Barcelona, Spain; ^2^Servicio de Urología, Instituto Valenciano de Oncología, C/Beltrán Báguena 8, 46009 Valencia, Spain

## Abstract

*Introduction*. The small renal masses (SRMs) have increased over the past two decades due to more liberal use of imaging techniques. SRMs have allowed discussions regarding their prognostic, diagnosis, and therapeutic approach. *Materials and methods.* Clinical presentation, incidental diagnosis, and prognosis factors of SRMs are discussed in this review. *Results.* SRMs are defined as lesions less than 4 cm in diameter. SRM could be benign, and most malignant SMRs are low stage and low grade. Clinical symptoms like hematuria are very rare, being diagnosed by chance (incidental) in most cases. Size, stage, and grade are still the most consistent prognosis factors in (RCC). An enhanced contrast SRM that grows during active surveillance is clearly malignant, and its aggressive potential increases in those greater than 3 cm. Clear cell carcinoma is the most frequent cellular type of malign SRM. *Conclusions.* Only some SRMs are benign. The great majority of malign SRMs have good prognosis (low stage and grade, no metastasis) with open or laparoscopic surgical treatment (nephron sparing techniques). Active surveillance is an accepted attitude in selected cases.

## 1. INTRODUCTION

The incidence of renal cell carcinoma (RCC) has increased over the past two decades reflecting earlier diagnosis at an earlier stage, largely due to more liberal use of radiological imaging techniques [1], introducing concepts as “incidental” or “small renal masses” (SRMs). SRM could be defined as those renal masses lower than 4 cm in diameter [2–4], accounting for 48–66% of RCC diagnosis [5]. Actually, 79–84% of SRM are detected before genitourinary symptoms are present [6–8] (size is smaller than symptomatic cancer classifying it as local stage with a better prognosis) [9]. Although mean tumor size has decreased in the last years, several studies indicate that this variable is one of the most important prognosis factors for RCC, and it has also contributed to the last modifications of RCC staging and treatment [10, 11].

Years ago, when most RCC were symptomatic, hematuria was the main symptom, so asymptomatic tumors were diagnosed later or not diagnosed. Before widespread use of imaging techniques, 67–74% of RCC remained undetected until death (autopsies), and only 8.9–20.0% of these undiagnosed RCC were responsible for the patient's death [5]. These data support the fact that some RCC have a favorable evolution and support active surveillance in select cases. Natural history of SRM has not been historically well established because most masses were surgically removed soon after diagnosis.

## 2. DEFINITIONS AND GENERAL CONCEPTS

A renal mass discovered by routine ultrasound, CT or MR indicated for other pathology, could be named incidental. A significant number of SRMs are incidentally diagnosed [2, 12]. Renal masses (benign and malign) can be considered incidental if they are diagnosed in the absence of symptoms or signs. “Incidentaloma” or “incidental” masses related to other organs such as adrenal, pituitary, thyroid and parathyroid, as well as the liver are published. Mirilas and Skandalakis questioned the scientific justification for this neologism and suggested that should be replaced by “incidentally found” [13]. Narrow relation of “incidental” and “small masses” are considered in some papers [2, 14–16]. A possible confusion factor may be that tumors classified as “incidental” show symptoms not directly attributable to the renal mass, thus not detected by the urologist [5].

Small renal masses include all solid or complex cystic lesions lower than 4 cm. Among them, different benign tumors are found in a 12.8 to 17.3% of cases [17–19] including oncocytoma in 53%, angiomyolipoma in 22%, atypical cyst in 10%, and different benign lesions as leiomyoma, xanthogranulomatous pyelonephritis, and focal infarction in 13% [17].

Incidental renal tumors have a mean size of 3.7 cm (median 3, range 0.8 to 12) [7]. Nevertheless, tumors greater than 4 cm could be incidental. Incidental diagnosis is performed in the 82.4%, 78.9%, and 56.7% of the 1–4 cm, 4–6 cm and greater than 6 cm renal masses, respectively [5]. If a cut-off should be made, most cases of RCC lower than 7 cm are incidentally discovered, while tumors greater than 7 cm are mainly symptomatic but, as mentioned previously, this cannot be taken as a rule [7].

## 3. SYMPTOMS

The main symptom of RCC is hematuria (35%–60%) [20–22] but SRMs are often asymptomatic (incidental). Classical manifestations of RCC such as fever or jaundice are extremely rare in front of an SRM. In a study of 349 SRM's, microhematuria was reported in only 8 cases. Prognostic of those RCC diagnosed by hematuria is worse than those incidentally diagnosed [23]. Stage I lesions were observed in 62.1% of patients with incidental RCC renal cell carcinoma and just in 23% with symptomatic RCC [6]. Among the different entities causing the incidental diagnosis of an SRM, many have been considered; evaluation for other malignancy (17.7%), gastrointestinal symptoms including nonspecific abdominal pain (16%), evaluation of medical renal disease (6.6%), hypertension (4%), back pain (5.1%), cirrhosis (1.4%), nephrolithiasis (1.4%), diverticulitis (1.4%), lung lesion (1.1%), increased liver enzymes (1.1%), trauma (0.8%), screening CT (0.8%), urinary tract infection (0.8%), chest pain (0.8%), aortic aneurysm evaluation (0.8%), cough (0.5%), shortness of breath (0.5%), Crohn's disease (0.5%), bronchocele (0.5%), and anemia (0.5%). No differences were found among incidental or symptomatic RCC according to age, sex, and laterality [15].

Laboratory findings have a significant impact on the patients with organ-confined RCC prognosis. Although, neoplasic condition reflects an increased invasive potential, characterized by overexpression of substances involved in cell proliferation as matrix metalloproteinases [24]; however, inflammatory markers like erythrocyte sedimentation rate greater than 30 mm/hour, hemoglobin levels less than 10 gm/dL (female) or 12 gm/dL (male), and increased alkaline phosphatase are negative prognosis elements [22].

Some demographic data may help to presume the matter of SRM: RCC is unusual in young patients; angiomyolipomas and multilocular cystic nephromas are more common in women [25].

## 4. PROGNOSIS FACTORS

Age is not a significant factor on survival in patients with incidental RCC [26], so it is probably not a prognosis factor for SRM [5]. However, as the patient ages, the SMR stage is higher; so the incidence of SRM finally staged as pT3 tumors in younger than 45 years, 45–75 years, and older than 75 years is 2.3%, 6.9%, and 14.3%, respectively [17]. The probability of developing metastases, with 12 years follow-up, is greater in men [27].

## 5. BENIGN TUMOR FREQUENCY

Lee et al. published 230 cases of SRM (lower than 4 cm), 88% malignant and 12% benign (oncocytoma) [6]. DeRoche et al. described that SRMs are nonneoplasic entities. Benign neoplasms and low-and high-grade carcinoma accounted for 1.6%, 18.0%, 49.0%, and 31.4%, respectively [8]. The percentage of malignancies increases from 72.1% in masses lower than 2 cm to 93.7% in tumors greater than 7 cm [7].

In conclusion, if the tumor is greater in dimensions, the possibility of being benign is lower; so tumors lower than 1, 2, 3, and 4 cm were benign in 46.3, 22.4, 22, and 19.9%, respectively [18].

## 6. SIZE AND STAGE

In a study from Schlomer et al., global mean renal tumor size decreased by 32% and pT1 tumors increased from 4% to 22% (1989–1998). For every cm increase in size, the odds ratio of malignancy increased 17–39% [7, 18]. Mean tumor size for benign tumors was 4.2 cm (median 3.3, range 0.2–25) compared to 6.3 cm (median 5.5, range 0.1–24) for malignant tumors. Median clinical diameter was 2.93 cm (range 0.8 to 4.0) in RCC lower than 4 cm. RCC mean size was 4.6 cm (range 0.8–21) and benign masses mean size 2.8 cm (range 0.8–9.5) [5]. Incidental RCC mean size was 3.7 cm (median 3, range 0.8–12) and symptomatic RCC mean size was 6.2 cm [7]. In pathological stage, 51.33% and 27.3% were pT1, 25.6% and 27.3% pT2, 10.9% and 23.8% pT3a, 10.9% and 16.6% pT3b, 1.2% and 2.3% pT3c, and 0% and 2.3% pT4 in incidental and symptomatic RCC, respectively.

Puppo et al. reported 94 patients with resected RCC (size: 1.1–4.5 cm), describing that pathological stage was pT1a in 92.5%, pT1b in 4.2%, and pT3a in 3.1% [28], similar to Pahernik et al. that reports pT1a in 84.5%, pT1b in 8%, and pT3 in 7.5% (organ confined in 92.5%) and ≥pT3 was found in 3.0%, 5.1%, and 12.1% of the patients when analyzed by tumor size 2, 3, and 4 cm, respectively [17]. A total of 25% of SRM doubled in volume within 12 months, 34% reached 4 cm and experienced rapid doubling time [5].

Kunkle et al. found synchronous metastatic disease increased by 22% with each cm increase in tumor size, by 50% for each increase of 2 cm, and doubled for each 3.5 cm increase in primary tumor size [11].

In other manuscript, incidental RCC had lower stages compared to symptomatic RCC [15]. Between T1a and T1b lesions, there was no significant difference in the rate of malignancy and high-grade malignancy regarding incidental or symptomatic presentation. The different percentage of T2 malignant tumors between incidental (90.9%) and symptomatic tumors was neither significant [5]. Understaging for pT3 tumors lower than 3 cm was 7.5% [17]. Cystic component appears in 24.1% of renal masses lower than 4 cm, being 57.1% in Bosniak type III and the rest in Bosniak type IV [5].

Volpe et al. showed no differences between the average growth rate for solid SRM (0.11 cm per year) and cystic masses (0.09 cm per year) [5]. Multifocality was present in 5.3–12% in small RCC [7, 8]. The rate of multifocality was 2.0%, 5.1%, and 7.05% in tumors of 2, 3, and 4 cm, respectively [17].

## 7. GRADE

Ninety percent of tumors lower than 1 cm were low-grade compared to only 37.9% of tumors ≥7 cm [18]. Grade 3 was found in 7.1%, 9.0%, and 14.0% of the patients in the 2, 3, and 4 cm groups, respectively and just 10.6% of small RCC were grade 3 [17]. Tumor grade increase as tumor size increase from 2 to 4 cm. Grade 1 was 31.3% for 2 cm, 27.4% for 3 cm, and 18.1% for 4 cm tumors; and grade 3 was 7.1% for 2 cm, 9% for 3 cm, and 14% for 4 cm tumors [17]. Urinary tract invasion, reported in some low-grade tumors, is a negative prognostic factor [29]. However, 45% of T2 incidental malignancies were high grade compared to 78.8% of T2 symptomatic malignancies [5]. Tumor grade increased according to size in clear cell, papillary, and chromophobe tumors. In high-grade carcinomas, 65% of the tumors had a 1-year volume doubling time.

## 8. CELLULAR TYPE

Clear cell is the most frequent cellular type regardless of tumor size [7]. Among SRM, Frank et al. showed that percentage of clear cell cellular type increased according to size: 59.9, 70.2, and 72% in lower than 2, 3, and 4 cm, respectively [18]. Cellular type for small RCC was 78% clear cell carcinoma, 15.3% papillary carcinoma, and 7% chromophobe carcinoma [17].

Volpe et al. showed that papillary RCC incidence is more frequent in 2 cm tumors than in 3 and 4 cm tumors (24%, 13.2%, and 13.5%, resp.) [17]; data not refuted by other authors [5]. Papillary cell type is more frequent than clear cell in tumors lower than 1 cm [18].

## 9. METASTASES

Metastases at diagnosis were found in 3.0%, 2.6%, and 6.0% of the patients with 2, 3, and 4 cm renal tumors, respectively [17]. Furthermore, lymph node spread was 4.8% and 15%, metastasis was 9.2% and 26%, and local recurrence was 1.2% and 8.3%, among incidental and symptomatic RCC, respectively [15]. With active surveillance, enhancing lesions with zero median growth rates did not progress to metastatic disease, and only 1.4% of patients with 0.31 cm yearly median growth rate progressed to metastatic disease [7]. Chawla et al. showed RCC mean growth rate of 0.40 cm yearly (median 0.35, range 0.42 to 1.6) [30].

Median tumor size for patients presented with pathologically confirmed synchronous metastatic disease was significantly greater than for those presenting with localized disease, 8.0 cm (range 2.2 to 20.0) and 4.5 cm (range 0.3 to 17.5), respectively. Tumors of 3.0 cm or smaller had synchronous metastasis in just 4.5% of the cases [31].

## 10. SURVIVAL

A total of 548 patients with small RCC were analyzed by Pahernik et al.: 22 (4%) had metastasis, 9 died by cancer in a mean time of 1.9 years (range 0.7 to 3.4) after diagnosis [17]. D’allOglio et al. observed a mean overall survival of 91% in patients with T1a tumors and up to 78.7% survival after 10 years of local treatment [15].

**Figure 1:**
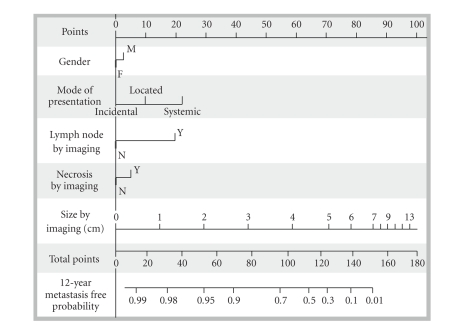
Preoperatory prognosis RCC nomogram [27].

Several groups have developed predictive models to construct prognosis algorithms in order to facilitate follow-up and to indentify progression risk. Raj et al. present a predictive model that includes gender, symptoms, radiological findings, and size as preoperative prognostic factors; in order to establish a chance of being cancer-free 12 years after surgery (Figure 1). In case of SRM, it could not be useful to decide surveillance or active treatment. For example, a woman with a 3 cm incidental malign SRM has a 96% chance of being cancer-free 12 years after surgery. In contrast, a man with a 4 cm symptomatic (local signs) malign SRM and positive TC showing enlarged lymph nodes has 60% chance of being cancer-free 12 years after surgery [27].

Classically, better prognosis has been assigned to incidental diagnosis, papillary or chromophobe pathology, small size, and early stage [32]. Presence of necrosis and vascular invasion is useful in a specific algorithm looked toward clear cell renal tumor [33].

Table 1 resumes the main prognosis factors useful on SRM.

## 11. TREATMENT AS PROGNOSIS FACTOR

Size is a significant factor in the decision to perform NSS: tumors sized 2 cm (81%), 3 cm (73%), and 4 cm (44%) cm could be treated by means of NSS. This treatment is technically easier in incidental than not incidental RCC (76% versus 24%) [15]. Local excision is a safe treatment for small RCC, even in extreme cases such as living donor kidney with a 5 × 5 mm RCC found on its surface [34]. In patients with RCC lower 4 cm, who underwent partial or radical nephrectomy 14% and 10% died during follow-up (cancer-specific death occurred in 3% in both approaches). Disease specific survival rate at 3 and 5 years is 95 and 97% in partial and radical nephrectomy, respectively [6].

When active surveillance is applied to 2 cm mean size contrast-enhancing renal masses, no differences were reported about age, sex, initial size, and solid versus cystic radiologic appearance. A significant different frequency of surgery was found among tumors with 0 or 0.31 cm mean yearly growth rate of 17% and 51%, respectively [7]. However, 33% of SRM under active surveillance showed zero or negative radiologic growth [7]. The probability to develop metastasis in masses lower than 3 cm managed by active surveillance was only 2% [14]. Prior and during follow-up, renal tumor biopsies are recommended. As a general rule, biopsy may be indicated in masses that have features of oncocytoma in poor surgical candidates. For patients who have a surgical contraindication or reject surgery, alternative ablation techniques can be proposed (cryoablation, radiofrequency) [35].

For Kassouf et al., 20.8% of renal masses showed tumor growth during the surveillance period (mean 31.6 months), but neither of them developed metastasis. Patients receiving surgical treatment after surveillance did not modify their prognostic [16]. Hereditary renal tumors may have a more aggressive natural history, and thus surveillance should be made with caution. Meta-analysis of Kunkle et al. observed no statistical differences in the incidence of SRM progression regardless excision, ablation, or active surveillance [2].

## 12. CONCLUSIONS

SRMs are those smaller than 4 cm, often incidentally diagnosed. Clinical symptoms, like hematuria, are rare, but confer worse prognosis. Size, stage, and grade are still the most consistent prognostic factors in RCC. It is important to keep in mind that SRM could be benign tumors, mainly oncocytoma. Most malign SMRs are low stage and low grade, without metastatic spread if diameter is below 2-3 cm. Clear cell carcinoma is the most frequent cellular type of malign SRM. Papillary tumors are more frequent when SRM size is less than 1 cm, having a better prognosis. Aggressive potential of small RCC could increase in tumors greater than 3 cm, so it is suggested that the threshold for selecting patients (old age, high-risk, solitary kidney, reject surgery) for a surveillance strategy should be set well below a tumor size of 3 cm. In active surveillance, the size increase of an SRM is a strong indicator of malignancy; helping to decide a surgical treatment.

## Figures and Tables

**Table 1:** Small RCC prognosis factors.

Better prognosis	Worse prognostic
Incidental	Symptoms
Small size <3 cm	Size > 3 cm
T1	T2 and >
Low grade	High grade
No upper tract invasion	Upper tract invasion
No lymph nodes	Necrosis
No necrosis	Lymph nodes
No vascular invasion	Vascular invasion
Negative biological markers	Positive biological markers
Papillary or chromophobe pathology (¿)	Sarcomatoid component
Zero median grown rate	Grown rate > 0.31 cm yearly
Option to NSS	No option to NSS
